# Robotic and standard surgical intervention as adjunct therapies for retroperitoneal ganglioneuroma resection: a case report

**DOI:** 10.1186/s12893-021-01146-x

**Published:** 2021-03-19

**Authors:** Wagner M. Tavares, Sabrina Araujo de Franca, Amsterdam S. Vasconcelos, David S. L. Parra, Sergio R. R. Araújo, Manoel J. Teixeira

**Affiliations:** 1 Department of Research of IPSPAC, Instituto Paulista de Saúde Para Alta Complexidade, 199 Padre Anchieta Avenue, Room 2, Jardim, Santo André, SP 09090-710 Brazil; 2 Institute of Neurology, University of São Paulo, 255 Dr. Enéas de Carvalho Aguiar avenue, Cerqueira César, São Paulo, SP 05403-900 Brazil; 3 Surgical Oncology Department, Hospital Santa Catarina, 200 Paulista Avenue, Bela Vista, São Paulo, SP 01310-000 Brazil; 4 LabPac, Laboratório Anatomia Patológica Imuno-Histoquímica Citopatologica, 75 Calixto da Mota Street, Vila Mariana, São Paulo, SP 04117-100 Brazil

**Keywords:** Adjunct surgery, Case report, Ganglioneuroma, Retroperitoneal, Robotic assisted surgery

## Abstract

**Background:**

Ganglioneuroma (GN) is ranked by the International Neuroblastoma Pathology Classification as a benign tumor. It can occur anywhere along the sympathetic nerve chain and surgical excision is the treatment of choice.

**Case presentation:**

An 18-year-old female patient sought medical assistance after 6 months of constant dorsal and back pain radiating from the thoracic region to the right abdominal flank. Magnetic resonance imaging revealed a solid nodular lesion with heterogeneous post-contrast enhancement and lobulated contours, centered on the right foramina of D12–L1, with a projection to the intracanal space, which compressed and laterally displaced the dural sac and had a right paravertebral extension between the vertebral bodies of D11 and superior aspect of L2. Ganglioneuroma was diagnosed using immunohistochemical analysis. It was decided to use a surgical approach in two stages: robot assisted for the anterior/retroperitoneal mass and a posterior hemilaminectomy/microsurgical approach to attempt total resection, avoiding the traditional anterior thoracoabdominal surgical incision and optimizing the patient’s postoperative outcomes. No postoperative adverse events were noted, and the patient was discharged on postoperative day 5.

**Conclusion:**

This retroperitoneal GN presentation was peculiar because it originated at the D12 nerve root, which extended to the retroperitoneal space and inside the spinal canal. We hope that our case report can assist future decisions in similar circumstances.

**Supplementary Information:**

The online version contains supplementary material available at 10.1186/s12893-021-01146-x.

## Background

Ganglioneuroma (GN) is the most mature form of a peripheral neuroblastic tumor [1, 2], i.e., an embryonic tumor of the sympathetic nervous system [2]. Histologically, it has a Schwannian rich stroma, with a low mitotic-karyorrhexis index, and few immature neuroblasts cells, which the International Neuroblastoma Pathology Classification [3, 4] ranks as histologically benign.

GNs can occur anywhere along the sympathetic nerve chain and commonly at the mediastinum, retroperitoneum, and adrenal glands [5]. Surgical excision is the treatment of choice.

Here, we describe the case of an 18-year-old female patient with retroperitoneal GN, which was surgically removed. The time elapsed between first consult and surgical intervention was four months. In addition to the tumor’s rarity, this case is of special interest because of the associated pathologic presentation of a retroperitoneal mass with spinal invasion and the innovative combination of robot-assisted surgery with a traditional neurosurgical approach.

## Case presentation

An 18-year-old female patient, with uneventful past medical history and no relevant family history, reported constant dorsal and back pain radiating from the thoracic region to the right abdominal flank for 6 months. Pain worsened at physical exertion. The pain was initially treated with anti-inflammatory drugs and analgesics, with a good response for 2 weeks, after which, it became progressively resistant to medication. The patient provided informed written consent that was approved by the IPSPAC Institutional Review Board.

### Imaging and clinical findings

Patients neurologic evaluation revealed decreased touch and pain perception between the T7 and T11 dermatomes. Reflexes, muscle strength, and laboratory results were normal.

**Fig. 1:**
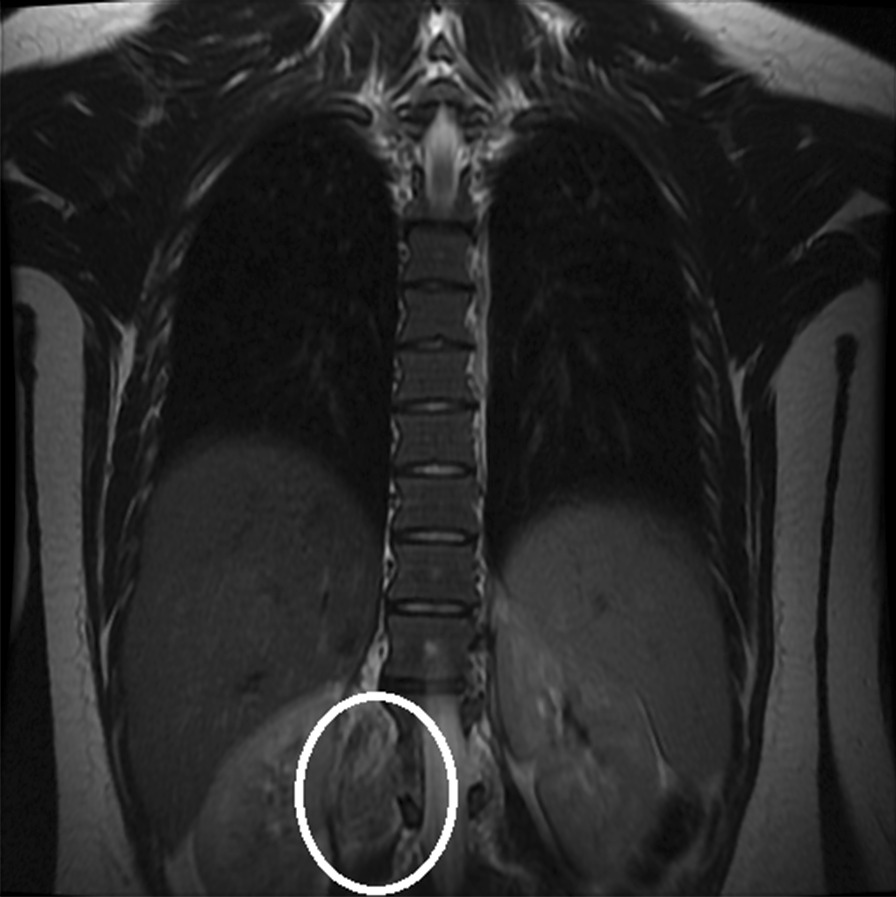
Coronal T2 magnetic resonance imaging. Circle: solid nodular lesion

**Fig. 2:**
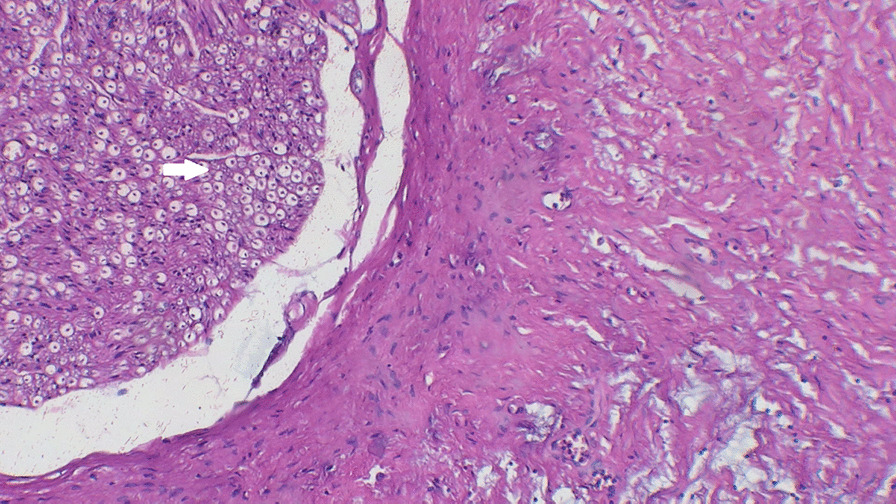
Fibrillar proliferation with a Schwannian pattern next to a nerve segment (arrow). Hematoxylin and eosin staining at 20 × magnification

**Fig. 3:**
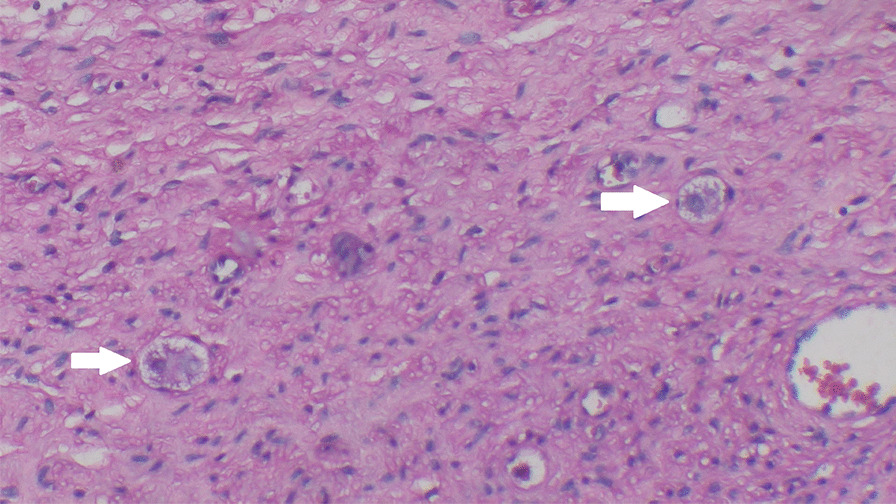
Ganglion cells (arrow). Hematoxylin and eosin staining at 40 × magnification. Superior long axis T2 fast spin echo imaging. Arrow: solid nodular lesion.

Gadolinium-enhanced spine magnetic resonance imaging (MRI) revealed a solid nodular lesion with heterogeneous post-contrast enhancement and lobulated contours centered on the right foramina of D12–L1, with a projection to the intracanal space, which compressed and laterally displaced the dural sac having a right paravertebral extension between the vertebral bodies of D11 and superior aspect of L2. It measured approximately 5.0 × 3.0 × 9.5 cm (latero-lateral × antero-posterior × caudocranial) (Fig. 1). Subsequently, the patient underwent guided biopsy with computed tomography that was histologically described as a focal fusocellular proliferation with dissociating skeletal-muscle fibers (Figs. 2, 3), and the immunohistochemical I19-1289 study confirmed it as a Schwannoma. However, as the MRI and immunohistochemical analysis [that was positive for S100 protein and (**SRY** (sex determining region Y)-**box 10**) (SOX-10) antigens] could suggest other diagnoses, we performed a lamina revision in a second pathology laboratory, which confirmed a diagnosis of GN.

After lamina revision, the patient quickly deteriorated with bladder dysfunction and worsening muscle strength on the proximal right lower limb, leading to an expedite surgical intervention conducted by neurosurgery and oncology team.

### Surgical procedure

Surgical intervention was planned through a combination of two approaches: robot assisted for the anterior/retroperitoneal stage and a posterior hemilaminectomy/microsurgical approach to attempt total resection.

The anterior access was performed as a right robot-assisted lateral transabdominal adrenalectomy using the da Vinci® Xi surgical system (Intuitive Surgical, Sunnyville, CA, USA). Preoperative preparation, patient positioning, and port-site access followed the lateral transabdominal laparoscopic adrenalectomy protocol. The patient was placed in the left lateral decubitus position and five trocars were inserted: an 8-mm camera port was inserted midway between the umbilicus and the right costal margin 20 cm away from the target; two robotic instrument ports, both 8 mm, were inserted along a line two fingerbreadths 8 cm away from each other over the umbilicus; one robotic instrument 8-mm port was inserted along the same line under the umbilicus at the right iliac pit away from the 8-cm camera port; a 5-mm liver retraction port was inserted in the midline in the epigastrium, and an accessory 10/12 mm auxiliary port was inserted between the camera port and right iliac pit port 7 cm away from each other. The four-step technique of robotic right adrenalectomy was applied [6]: (1) complete division of the hepatocolic ligament; (2) delineation of the right adreno-caval junction; (3) division of the right adrenal vein; (4) adrenal separation from the kidney, retroperitoneal approach in retrocaval space dissection and identification of the tumor circumferentially. Thus, after first robotic exploration, the triangular ligament was divided via a robotic monopolar hook. The right lobe of the liver was retracted with a laparoscopic retractor (Additional file 1: Video S1), and the inferior vena cava was exposed after the inferior to superior peritonectomy. The surgeon at the console used the robotic hook for precise dissection of the vena cava along its lateral edge between the inferior vena cava and liver. Landmarks identified included laterally the superior pole of the right kidney and posteriorly the psoas muscle. After careful dissection (Additional file 2: Video S2), a yellowish, fibroelastic, well encapsulated mass was identified adjacent to the periadrenal space. It lousily adhered to the adjacent structures and could be thoroughly isolated. The surgeon performed the laparoscopic suturing of the diaphragm (Additional file 3: Video S3) before fully dissect the anterior portion, which was successful until the right limit of the D12 vertebra.

**Fig. 4:**
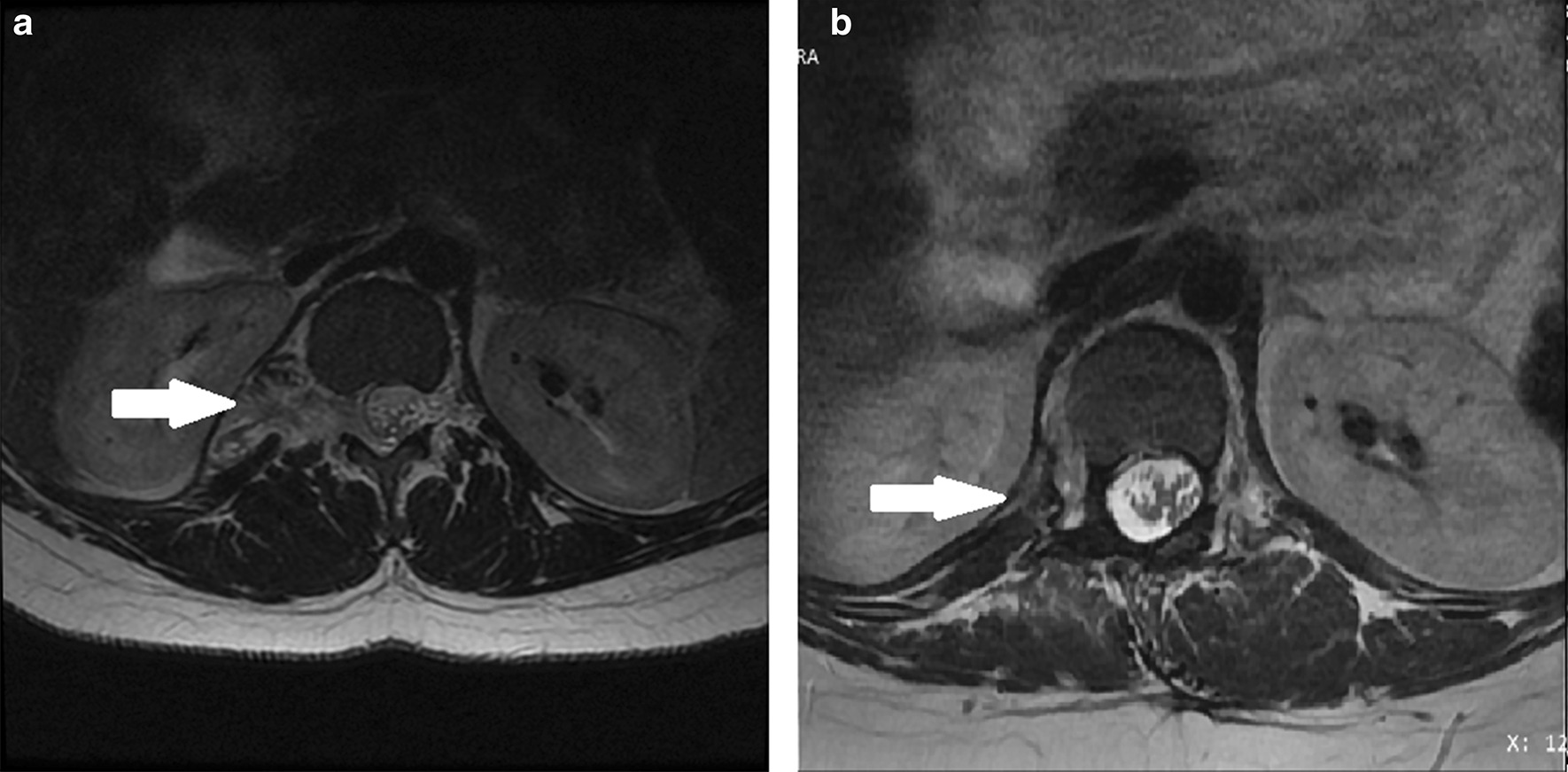
Superior long axis T2 fast spin echo imaging. Arrow: solid nodular lesion. **a** Before surgery. **b** After surgery

Afterward the anterior step was performed, the patient was positioned in ventral decubitus position and prepared for a traditional laminectomy. After a dorsolumbar medial incision, a regular bilateral paraspinal muscular dissection was performed. The D11, D12, and L1 lamina, facets, and transverse process were exposed. After performing a D11–L1 hemilaminectomy, the tumor’s origin was identified from the D12 nerve root. The tumor extended from the retroperitoneal space to the spinal canal, entering from the D12–L1 right foramina and compressing the dura mater. Following the reclamation of the D11–D12 zygapophyseal facet and D12 transverse process, the posterior intracanal and paravertebral elements of the mass were removed under microscope visualization. The D12 nerve root had to be severed for total tumor removal; afterward, the retroperitoneal space could be recognized. Postoperative pathologic examination confirmed that it was a GN. Figure 4 demonstrates the patient before and after tumor removal.

### Postoperative course

Postoperatively, the patient recovered well. On postoperative day 1, the patient experienced episodes of muscle pain in the abdominal wall and laminectomy region, controlled with analgesics and no further investigation was needed. Patient mobilization occurred on postoperative day 2, when the patient could walk with assistance. The patient was discharged on postoperative day 5. After 1-year of follow-up, the patient had improved muscle strength but still experienced episodes of myofascial pain in the dorsum and on the right lateral abdominal flank, again controlled with analgesics. However, she did not adhere to her physical rehabilitation protocol.

## Discussion and conclusions

GNs are rare benign neurogenic (ganglion cell) tumors, commonly affecting adolescents and youth adults [7, 8]. Although GNs usually develop in childhood, adult detection can be explained by their slow asymptomatic growth [9–11], justifying the delay in seeking medical assistance [12].

Retroperitoneal GN commonly presents local mass-effect symptoms, leading to an often casual diagnosis [5, 11, 13]. In our case, the patient sought medical assistance because of secondary symptoms (dorsal and back pain radiating to the abdominal flank), explained by the local mass-effect compression at D12–L1, which laterally displaced the dural sac, decreasing touch and pain perception between the T7 and T11 dermatomes.

GN is also distinguished by the increased production of catecholamines, with Verner–Morrison syndrome as the recurrent example of this endocrine dysregulation.[8, 12, 14, 15] However, this phenomenon rarely emerges in mature GNs [9].

Cai et al. [16] reported on 17 patients with retroperitoneal GN, of whom, 13 did not present obvious clinical symptoms or signs. Four patients had palpable masses, hypertension, or diarrhea. Our patient did not mention or presented with symptoms of diarrhea, sweating, or hypertension, leading us to conclude that this could be a mature GN. Later bladder dysfunction is also observed in lower thoracic or lumbar GNs [12].

Preoperative diagnosis predominantly relies on imaging techniques. Reported imaging findings [7, 8, 17] include extensive calcifications with most cases being well-circumcised nondescript homogenous masses, similar to our case. Calcifications are secondary specific histologic features of neuroblastic tumors [2, 18], being present in up to 60% of cases [19, 20]. In GNs, calcification is normally discrete and punctuate, different from that in neuroblastomas (coarse, amorphous, and mottled) and ganglioneuroblastomas (granular) [21]. Careful examination of all laminas is necessary to avoid overlooking small calcification foci [2].

Considering the indefinite diagnosis suggested by the imaging examination, a biopsy and/or excision for histopathological examination is frequently performed. Besides the level of cellular maturity, distinct between malignant neuroblastic (neuroblastoma and ganglioneuroblastoma) tumors and GNs is the presence of hemorrhage, metastasis, and necrosis in malignant presentations [22].

The International Neuroblastoma Pathology Committee [2] standardized the criteria for GN histologic diagnosis, defined by the predominant composition of a ganglioneuromatous stroma with an insignificant component of mature ganglion cells (Fig. 4). For such anatomic sites, GN should be the diagnosis of choice, because of the neurofibroma-like lesion, whether or not ganglion cells are identified [7]. In the retroperitoneum, Schwannoma cells are usually more cellular, with larger plump spin cells, showing diffused and strong expression of S-100 protein [7, 17], which was present in our patient’s histopathological results. Likewise, greater expression of SOX-10, which is correlated with glial differentiation, has been found in GNs [23]. Our histopathological specimen was pre- and postoperatively positive for SOX-10.

Although partial or no resection can be accepted as a GN treatment [24, 25], the long-term prognosis is improved with total resection, and recurrence surveillance can be made through image control [10, 26]. In our case, total resection was achieved by association of two surgical techniques: robot-assisted lateral transabdominal and posterior laminectomy. During surgical excision, it was noticed that the tumor originated from the nerve root, which is unusual for GNs with only few cases described in the literature [10, 24, 25, 27, 28].

Nevertheless, to our knowledge, no other case treatment was as innovative as the association of these two surgical techniques. The traditional option for removing a retroperitoneal mass would involve an extensive thoracoabdominal surgical incision, such as in adrenalectomy procedures. In such cases, open surgery can result in higher doses of analgesics [29–31], greater blood loss [30, 32], increased hospital stay [29–33], and delay in return to normal activities [29, 31–33]. The combined robot-assisted method was selected because of the mass size and presumed benignancy, offering more advantages regarding invasiveness and postoperative recovery outcomes. We hope that our case report can assist future decisions in similar circumstances.

## Supplementary Information

**Additional file 1: Video S1.** Video that demonstrates liver retraction.mp4.

**Additional file 2: Video S2.** Video that demonstrates tumor dissection.mp4

**Additional file 3: Video S3.** Video that demonstrates laparoscopic suturing of the diaphragm.mp4.

## Data Availability

Data and materials are available upon request by contacting the corresponding author Sabrina Araujo de Franca. ORCID: 0000-0003-2682-4537, IPSPAC—Instituto Paulista de Saúde para a Alta Complexidade, 199 Padre Anchieta Avenue—Room 2, Jardim, Santo Andre, SP, 09090-710, Brazil; E-mail: pesquisacientifica@ipspac.org.br; Telephone: + 1 (514) 804-1006.

## Abbreviations

CM: Centimeter
GN: Ganglioneuroma
MM: Millimeter
MRI: Magnetic resonance imagining
SRY: Sex determining region Y
SOX-10: SRY-box 10
